# Surgical outcomes of lung cancer associated with autoimmune disease-related interstitial pneumonia

**DOI:** 10.1007/s11748-025-02229-9

**Published:** 2025-11-21

**Authors:** Gouji Toyokawa, Haruaki Hino, Takaki Akamine, Mototsugu Shimokawa, Masaaki Sato

**Affiliations:** 1 Department of Thoracic Surgery, The University of Tokyo Hospital, 7-3-1 Hongo, Bunkyo-ku, Tokyo, 113-8655 Japan; 2 Department of Surgery and Science, Graduate School of Medical Sciences, Kyushu University, Fukuoka, Japan; 3 Department of Biostatistics, Graduate School of Medicine, Yamaguchi University, Yamaguchi, Japan

**Keywords:** Lung cancer, Interstitial pneumonia, Autoimmune disease, Surgery, Acute exacerbation of interstitial pneumonia

## Abstract

**Objective:**

Generally, lung cancer originating from interstitial pneumonia (IP) is considered more aggressive; however, lung cancer in patients with autoimmune disease (AD)-related IP (AD-IP) is not well documented. This study aimed to clarify surgical outcomes and the risk of postoperative acute exacerbation (AE) in patients with lung cancer associated with AD-IP.

**Methods:**

We retrospectively reviewed patients with lung cancer who underwent surgery between January 2011 and March 2021. Clinicopathological characteristics, recurrence-free survival (RFS), overall survival (OS), and perioperative outcomes were compared according to the presence of IP with or without AD.

**Results:**

Among 1281 patients with lung cancer, 61 (4.8%) had idiopathic interstitial pneumonia (IIP), 87 (6.8%) had AD without IP, and 26 (2.0%) had AD-IP. The 5-year RFS rates were 80.9% for patients without IIP or AD, compared with 48.0% for IIP, 76.1% for AD without IP, and 29.8% for AD-IP. The corresponding 5-year OS rates were 84.1%, 53.9%, 77.0%, and 34.5%. Patients with AD-IP were significantly younger (*P* = 0.001), were more often female (*P* < 0.001), had a lower % vital capacity (*P* = 0.002), and more frequently received preoperative steroids (*P* < 0.001). The overall incidence of AE among the 87 patients with IP was 10.3% (9/87): 9.8% (6/61) with IIP and 11.5% (3/26) with AD-IP, with no significant difference.

**Conclusions:**

Lung cancer with AD-IP had a poor prognosis, as did lung cancer with IIP, and the frequency of postoperative AE of patients with AD-IP was as high as that in those with IIP.

**Supplementary Information:**

The online version contains supplementary material available at 10.1007/s11748-025-02229-9.

## Introduction

Generally, lung cancer associated with interstitial pneumonia (IP) is biologically more aggressive than lung cancer without IP. A large-scale Japanese study showed that among patients with stage IA lung cancer, the 5-year overall survival (OS) rates in those with any type of IP and those without IP were 59.0% and 86.8%, respectively [1]. Importantly, despite recent advances in radiation, molecular-targeted therapies, and immune checkpoint inhibitors, most patients with IP cannot receive these treatments because of the risk of acute exacerbation (AE) of IP (IP-AE). This underscores the central role of surgery in managing IP-related lung cancer. However, the major challenge lies in the risk of postoperative IP-AE, which occurs in 9.3% of patients within 30 days of surgery and carries a mortality rate of 43.9% [2].

Autoimmune disease (AD) is also recognized as a risk factor for lung cancer, with a reported frequency of approximately 10% [3, 4]. We recently showed that among patients with lung cancer, OS and recurrence-free survival (RFS) rates were significantly lower in those with AD than without AD after surgical resection [4]. Other studies have also reported poorer outcomes in patients with lung cancer with than without AD [5, 6]. Additionally, AD frequently coexists with IP, occurring in approximately 10% of patients with IP [7]. The 1-year incidence of AE in AD-related IP (AD-IP) has been reported to range from 1.3 to 3.3% after diagnosis [8, 9]. However, treatment outcomes and the true frequency of postoperative IP-AE in patients with AD-IP associated lung cancer remain unclear.

The present study was performed to compare the clinicopathological features, perioperative outcomes including IP-AE, and postoperative survival between patients with lung cancer who had AD-IP and those who had idiopathic interstitial pneumonia (IIP).

## Methods

### Study cohort

Between January 2011 and March 2021, 1346 patients underwent surgery for lung cancer at The University of Tokyo Hospital. Thirty-seven patients with clinical, surgical or pathological stage IV disease were excluded. An additional 28 patients were excluded because of insufficient or unclear clinicopathological and perioperative data—including surgical procedure, histology, and pathological stage. As a result, 1281 patients were included in the present study. Clinicopathological characteristics collected included age at surgery, sex, smoking history, surgical procedure, histological type, and pathological tumor-node-metastasis (TNM) stage (according to the 7th edition of the lung cancer staging system). After pulmonary resection, routine follow-up consisted of physical examinations, blood tests, and chest X-rays every 3 months for the first 2–3 years, followed by every 6 months thereafter. Computed tomography (CT) scans were performed twice yearly during the first 5 years, and then at least annually up to 10 years after surgery. Follow-up data were collected until March 2025. This study was approved by the Ethics Committee of The University of Tokyo Hospital (IRB#: 2406-[11]).

### Diagnosis of IP and AE

Subtypes of IP were diagnosed by a multidisciplinary discussion (MDD) of physical, serological, high-resolution CT (HRCT), and pathological findings after surgical resection, in accordance with the official statement on IIPs [10, 11]. The diagnosis of AD was confirmed by specialists based on physical and serological findings, and patients with AD whose HRCT or pathological findings were consistent with IP were classified as having AD-IP [12]. Other pulmonary diseases were excluded by examination of the resected lung specimens. Postoperative AE following lung resection was defined based on previous reports [13]. Briefly, the six criteria for AE were as follows: onset within 30 days after pulmonary resection; intensified dyspnea; an increase in interstitial shadows on both chest radiographs and CT scans; a decrease in arterial oxygen tension of more than 10 mmHg under comparable conditions; no evidence of pulmonary infection; and exclusion of alternative causes such as cardiac failure, pulmonary embolism, or other identifiable causes of lung injury. Data on postoperative IP-AE and risk scores for IP-AE were also collected [14].

### Statistical analysis

Categorical variables were summarized as numbers and percentages, and continuous variables were presented as median values with the first and third interquartile ranges (IQR). RFS was defined as the interval from the date of surgery to the date of recurrence, death, or last follow-up. OS was defined as the interval from the date of surgery to the date of death from any cause or the last follow-up. Associations between groups and continuous variables were analyzed using the Mann–Whitney or Kruskal–Wallis test, while associations with categorical variables were evaluated using the chi-square test. Survival probabilities were estimated with the Kaplan–Meier method, and differences between survival curves were assessed using the log-rank test. A *P*-value of < 0.05 was considered statistically significant. All analyses were performed using JMP® 18.0 (SAS Institute, Cary, NC, USA) and Prism 8.0 (GraphPad Software, San Diego, CA, USA).

## Results

### Patient characteristics

The patient characteristics for the present study are shown in Table 1. Among them, 61 (4.8%) had IIP, 87 (6.8%) had AD without IP, and 26 (2.0%) had AD-IP. Among AD patients without IP, rheumatoid arthritis was the most common (17 patients [19.5%]), followed by Hashimoto’s disease (11 [12.6%]), whereas among those with AD-IP, systemic sclerosis was the most common (9 [34.6%]) followed by rheumatoid arthritis (8 [30.8%]). Further detailed information on AD without IP and AD-IP is provided in Supplementary Table 1.

**Table 1:** Clinicopathological characteristics of patients in the present study and comparisons between patients with IIP, AD without IP, AD‑IP, and those without these conditions

Characteristics		Total population (n = 1281)	IIP (n = 61)	AD without IP (n = 87)	AD-IP (n = 26)	Without any (n = 1107)	*P-*value
Age (years)	Median (IQR)	71 (64, 76)	75 (70, 79)	70 (65, 74)	67 (61, 74)	71 (64, 76)	< 0.001
Sex	Female	518 (40.4%)	7 (11.5%)	51 (58.6%)	13 (50.0%)	447 (40.4%)	< 0.001
	Male	763 (59.6%)	54 (88.5%)	36 (41.4%)	13 (50.0%)	660 (59.6%)	
BMI (kg/m^2^)	Median (IQR)	22.4 (20.2, 24.7)	23.2 (21.1, 24.8)	21.5 (19.5, 24.5)	22.4 (20.5, 24.3)	22.4 (20.2, 24.8)	0.041
Smoking history	Never	457 (35.6%)	7 (11.5%)	32 (36.8%)	5 (19.2%)	411 (37.2%)	0.002
	Ex or current	824 (64.4%)	54 (88.5%)	55 (63.2%)	21 (80.8%)	694 (62.8%)	
Brinkman index	Median (IQR)	470 (0, 1000)	940 (711, 1485)	400 (0, 1020)	775 (275–980)	400 (0, 1000)	< 0.001
FEV1.0 (mL)	Median (IQR)	2170 (1770, 2630)	2380 (1910, 2745)	1970 (1630, 2440)	1955 (1435, 2330)	2180 (1780, 2670)	< 0.001
FEV1.0% (G)	Median (IQR)	73.0 (66.5, 78.4)	74.3 (67.7, 79.1)	74.0 (67.0, 78.3)	76.1 (69.2, 80.5)	72.8 (66.1, 78.3)	0.324
VC (mL)	Median (IQR)	3060 (2530, 3700)	3350 (2695, 3885)	2790 (2245, 3505)	2660 (1895, 3015)	3100 (2570, 3710)	< 0.001
%VC	Median (IQR)	101.2 (90.9, 112.9)	99.0 (89.5, 110.9)	102.0 (83.6, 114.0)	86.0 (68.8, 106.0)	102.0 (91.3, 112.9)	0.002
Surgical procedure	Wedge resection	219 (17.1%)	13 (21.3%)	16 (18.4%)	7 (26.9%)	183 (16.5%)	0.249
	Segmentectomy	106 (8.3%)	1 (1.6%)	10 (11.5%)	1 (3.9%)	94 (8.5%)	
	Lobectomy, bilobectomy or pneumonectomy	952 (74.3%)	47 (77.1%)	60 (69.0%)	18 (69.2%)	826 (74.6%)	
	Others	4 (0.3%)	0 (0.0%)	1 (1.1%)	0 (0.0%)	4 (0.4%)	
Histology	Adenocarcinoma	954 (74.4%)	28 (45.9%)	65 (74.7%)	13 (50.0%)	848 (76.6%)	< 0.001
	Squamous cell carcinoma	227 (17.7%)	20 (32.8%)	17 (19.5%)	9 (34.6%)	181 (16.4%)	
	Others	100 (7.8%)	13 (21.3%)	5 (5.8%)	4 (15.4%)	78 (7.0%)	
Pathological stage	0	69 (5.4%)	2 (3.3%)	5 (5.8%)	0 (0.0%)	62 (5.6%)	0.003
	I	924 (72.1%)	35 (57.4%)	63 (72.4%)	15 (57.7%)	811 (73.3%)	
	II	164 (12.8%)	11 (18.0%)	13 (14.9%)	4 (15.4%)	136 (12.3%)	
	III	124 (9.7%)	13 (21.3%)	6 (6.9%)	7 (26.9%)	98 (8.8%)	
ly*	Absent	1036 (81.5%)	33 (54.1%)	72 (83.7%)	16 (61.5%)	915 (83.3%)	< 0.001
	Present	236 (18.5%)	28 (45.9%)	14 (16.3%)	10 (38.5%)	184 (16.7%)	
v*	Absent	790 (62.1%)	18 (29.5%)	56 (65.1%)	10 (38.5%)	706 (64.2%)	< 0.001
	Present	482 (37.9%)	43 (70.5%)	30 (34.9%)	16 (61.5%)	393 (35.8%)	
pl**	Absent	960 (75.5%)	36 (59.0%)	66 (77.6%)	11 (42.3%)	847 (77.1%)	< 0.001
	Present	311 (24.5%)	25 (41.0%)	19 (22.4%)	15 (57.7%)	252 (22.9%)	
Postoperative survival	Alive	1003 (78.3%)	36 (59.0%)	63 (72.4%)	10 (38.5%)	894 (80.8%)	< 0.001
	Dead	278 (21.7%)	25 (41.0%)	24 (27.6%)	16 (61.5%)	213 (19.2%)	

*AD* autoimmune disease, *BMI* body mass index, *FEV1.0* forced expiratory volume in 1 s, *IIP* idiopathic interstitial pneumonia, *IP* interstitial pneumonia, *IQR* interquartile range, *VC* vital capacity

*Data were missing for 9 patients

**Data were missing for 10 patients

### Comparison of patient characteristics, perioperative outcomes, and survival following surgery among four groups

**Fig. 1:**
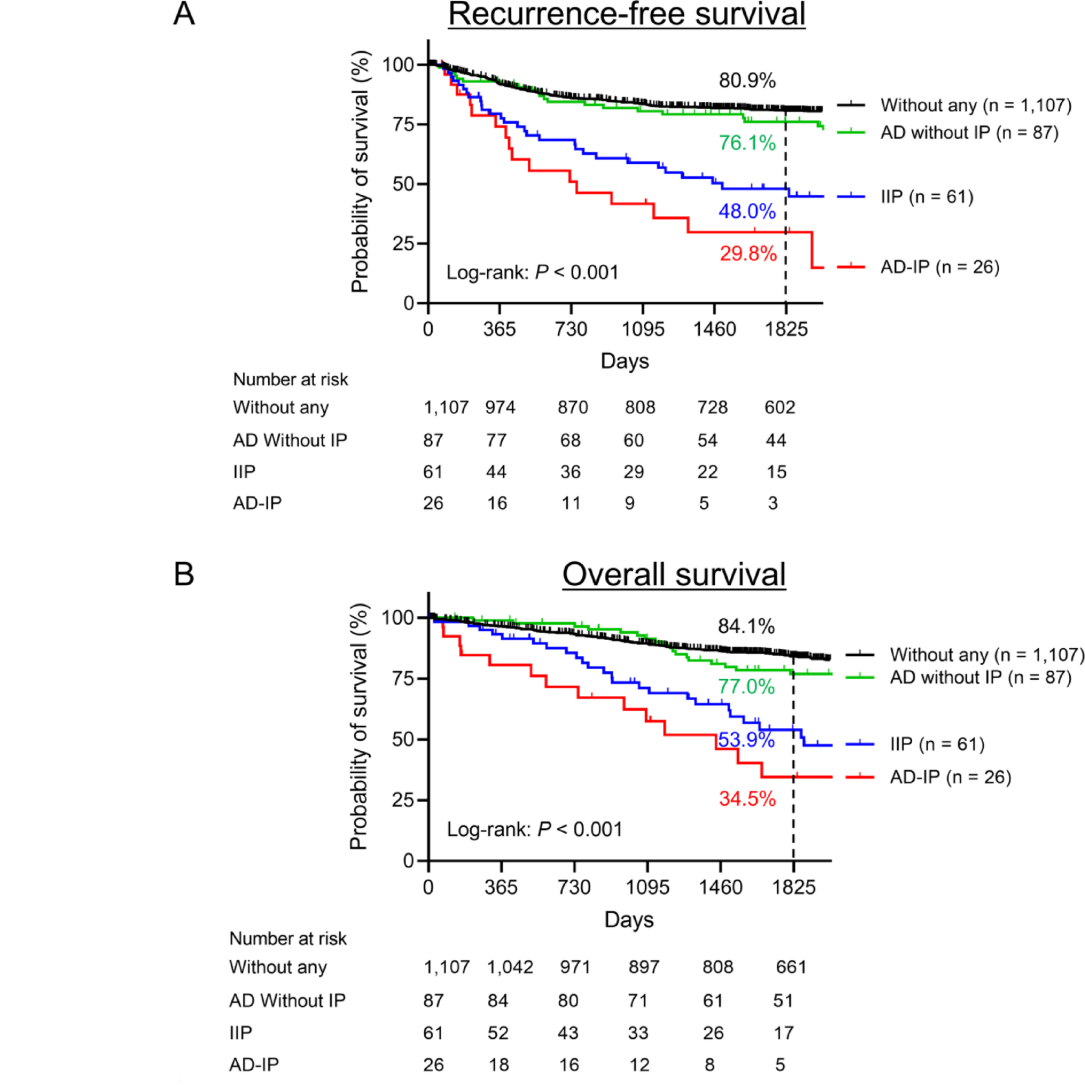
Survival following surgery among patients with lung cancer. **A** Recurrence-free survival and **B** overall survival of patients with IIP, AD without IP, and AD-IP and patients without these conditions (log-rank test: *P* < 0.001 for both). AD, autoimmune disease; AD-IP, autoimmune disease–related interstitial pneumonia; IIP, idiopathic interstitial pneumonia; IP, interstitial pneumonia

A comparison across the four groups in patient characteristics is provided in Table 1. Perioperative outcomes of each group are also shown in Supplementary Table 2. Both IIP and AD-IP were significantly associated with more pathologically malignant features and longer postoperative hospital stays. The median follow-up period was 1987 days (first, third IQR 1229 days, 2959 days). The 5-year RFS and OS rates for the entire cohort of 1281 patients with lung cancer were 80.9% and 84.1%, respectively (Supplementary Figs. 1A and B). The 5-year RFS rates were 80.9% for patients without IIP or AD, compared with 48.0% for IIP, 76.1% for AD without IP, and 29.8% for AD-IP (*P* < 0.001) (Fig. 1A). The corresponding 5-year OS rates were 84.1%, 53.9%, 77.0%, and 34.5%, respectively (*P* < 0.001) (Fig. 1B). The lung cancer specific 5-year OS rates were 91.5%, 62.9%, 90.4%, and 47.9%, respectively (*P* < 0.001) (Supplementary Fig. 2). The RFS, OS, and lung cancer-specific survival rates did not differ significantly between the IIP and AD-IP groups (*P* = 0.317, 0.085, and 0.134, respectively).

### Comparison of patient characteristics and perioperative outcomes between IIP and AD-IP

Patients with AD-IP were significantly younger (*P* = 0.001), were more likely to be female (*P* < 0.001), had a lower Brinkman index (*P* = 0.022), had a lower forced expiratory volume in 1 s (*P* = 0.004), had a lower vital capacity (VC) (*P* = 0.002), had a lower %VC (*P* = 0.002), and had a lower diffusing capacity of the lung for carbon monoxide (*P* = 0.046) (Table 2). Regarding the radiological findings of IP, 11 (18.3%) patients in the IIP group and 8 (30.8%) patients in the AD-IP group exhibited a usual IP pattern, with no significant differences observed between the two groups (*P* = 0.271). Seventeen (68.0%) patients in the IIP group and 11 (68.8%) patients in the AD-IP group died of lung cancer recurrence, while 3 (12.0%) and 2 (12.5%) patients died of AE of IP, respectively. No patients in the AD-IP group died of AD. With respect to perioperative outcomes, no significant differences were observed between IIP and AD-IP (Table 3).

**Table 2:** Comparison of clinicopathological characteristics of patients with IIP and AD-IP

Characteristics		IIP (n = 61)	AD-IP (n = 26)	*P-*value
Age (years)	Median (IQR)	75 (70, 79)	67 (61, 74)	0.001
Sex	Female	7 (11.5%)	13 (50.0%)	< 0.001
	Male	54 (88.5%)	13 (50.0%)	
BMI (kg/m^2^)	Median (IQR)	23.2 (21.1, 24.8)	22.4 (20.5, 24.3)	0.361
Smoking history	Never	7 (11.5%)	5 (19.2%)	0.337
	Ex or current	54 (88.5%)	21 (80.8%)	
Brinkman index	Median (IQR)	940 (711, 1485)	775 (275–980)	0.022
FEV1.0 (mL)	Median (IQR)	2380 (1910, 2745)	1955 (1435, 2330)	0.004
FEV1.0% (G)	Median (IQR)	74.3 (67.7, 79.1)	76.1 (69.2, 80.5)	0.401
VC (mL)	Median (IQR)	3350 (2695, 3885)	2660 (1895, 3015)	0.002
%VC (%)	Median (IQR)	99.0 (89.5, 110.9)	86.0 (68.8, 106.0)	0.009
DLCO’ (%)*	Median (IQR)	86.3 (70.7, 98.2)	81.6 (55.9, 89.5)	0.046
Radiological findings of IP**	UIP	11 (18.3%)	8 (30.8%)	0.271
	Probable UIP	23 (38.3%)	11 (42.3%)	
	Indeterminate or alternative	26 (43.4%)	7 (26.9%)	
Surgical procedure	Wedge resection	13 (21.3%)	7 (26.9%)	0.842
	Segmentectomy	1 (1.6%)	1 (3.9%)	
	Lobectomy, bilobectomy or pneumonectomy	47 (77.0%)	18 (69.2%)	
Histology	Adenocarcinoma	28 (45.9%)	13 (50.0%)	0.814
	Squamous cell carcinoma	20 (32.8%)	9 (34.6%)	
	Others	13 (21.3%)	4 (15.4%)	
Pathological stage	0	2 (3.3%)	0 (0.0%)	0.759
	I	35 (57.4%)	15 (57.7%)	
	II	11 (18.0%)	4 (15.4%)	
	III	13 (21.3%)	7 (26.9%)	
ly	Absent	33 (54.1%)	16 (61.5%)	0.522
	Present	28 (45.9%)	10 (38.5%)	
v	Absent	18 (29.5%)	10 (38.5%)	0.413
	Present	43 (70.5%)	16 (61.5%)	
pl	Absent	36 (59.0%)	11 (42.3%)	0.152
	Present	25 (41.0%)	15 (57.7%)	
Postoperative survival	Alive	36 (59.0%)	10 (38.5%)	0.079
	Dead	25 (41.0%)	16 (61.5%)	
Cause of postoperative death	Lung cancer	17 (68.0%)	11 (68.8%)	0.995
	AE of IP	3 (12.0%)	2 (12.5%)	
	Others	5 (20.0%)	3 (18.8%)	

*AD* autoimmune disease, *AE* acute exacerbation, *BMI* body mass index, *DLCO* diffusing capacity of the lung for carbon monoxide, *FEV1.0* forced expiratory volume in 1 s, *IIP* idiopathic interstitial pneumonia, *IP* interstitial pneumonia, *IQR* interquartile range, *UIP* usual interstitial pneumonia, *VC* vital capacity

*Data were missing for 2 patients

**Data were missing for 1 patient

**Table 3:** Comparison of perioperative outcomes in patients with IIP and AD-IP

Characteristics		IIP (n = 61)	AD-IP (n = 26)	*P-*value
Postoperative hospital stay (days)	Median (IQR)	10 (7, 14)	11 (8, 18)	0.144
Operation time (min)	Median (IQR)	185 (120, 242)	152 (105, 242)	0.576
Blood loss (mL)	Median (IQR)	100 (25, 216)	110 (0, 165)	0.572
Re-operation	N (%)	2 (3.3%)	3 (11.5%)	0.130
Cardiovascular complications	N (%)	2 (3.3%)	0 (0.0%)	0.350
Bronchopulmonary fistula	N (%)	0 (0.0%)	1 (3.8%)	0.123
Pneumonia	N (%)	5 (8.2%)	5 (19.2%)	0.140
Empyema	N (%)	3 (4.9%)	1 (3.9%)	0.827
Prolonged air leak	N (%)	11 (18.0%)	3 (11.5%)	0.451
AE of IP	N (%)	6 (9.8%)	3 (11.5%)	0.811
Postoperative death within 30 days	N (%)	1 (1.64%)	0 (0.0%)	0.511

*AD* autoimmune disease, *AE* acute exacerbation, *IIP* idiopathic interstitial pneumonia, *IP* interstitial pneumonia, *IQR* interquartile range

### Comparison of IP-AE in patients with IIP and AD-IP

**Fig. 2:**
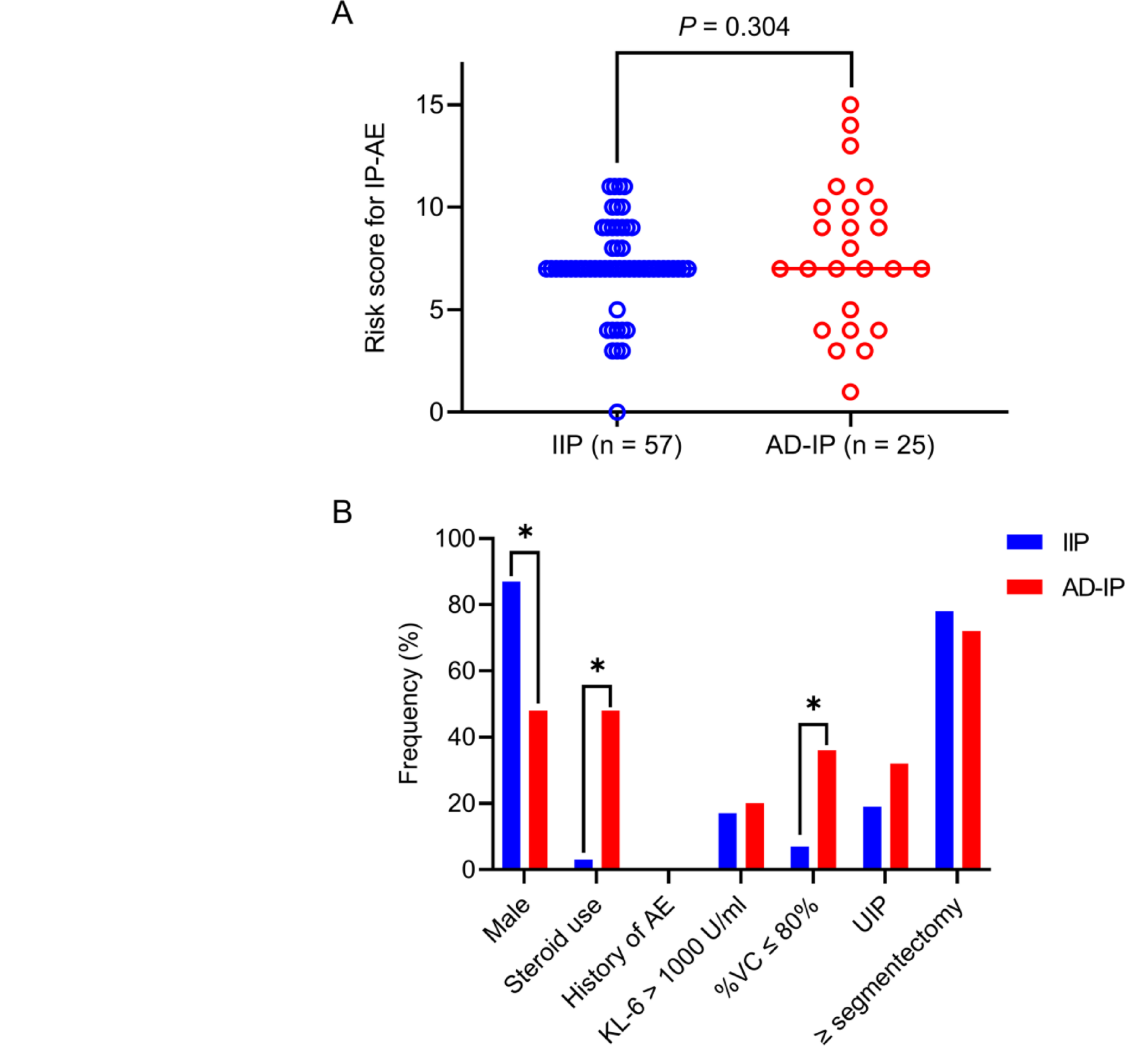
Comparison of IP-AE risk score between IIP and AD-IP. **A** Risk score for IP-AE in IIP and AD-IP (median values: 7 vs. 7; *P* = 0.304). **B** Distribution of each variable included in the IP-AE risk score, comparing IIP and AD-IP (*P < 0.001). AD-IP, autoimmune disease–related interstitial pneumonia; AE, acute exacerbation; IIP, idiopathic interstitial pneumonia; IP-AE, acute exacerbation of interstitial pneumonia

Data on risk factors for IP-AE were missing in 4 of the 61 patients with IIP and in 1 of the 26 with AD-IP. Although the median risk score for IP-AE did not differ significantly between IIP and AD-IP (7 vs. 7; *P* = 0.304) (Fig. 2A), AD-IP was significantly associated with female sex (*P* < 0.001), preoperative steroid use (*P* < 0.001), and %VC of ≤ 80% (*P* < 0.001) compared with IIP (Table 4 and Fig. 2B). Among the 12 patients who received preoperative steroid therapy, 1 patient received 5 mg of methylprednisolone, 1 patient received 15 mg of hydrocortisone, 1 patient received 1 mg of prednisolone, 1 patient received 2.5 mg of prednisolone, 5 patients received 5 mg of prednisolone, 1 patient received 10 mg of prednisolone, 1 patient received 15 mg of prednisolone, and 1 patient received 20 mg of prednisolone. The incidence of AE among the 87 patients with lung cancer and IP was 10.3% (9/87): 9.8% (6/61) in those with IIP and 11.5% (3/26) in those with AD-IP, with no significant difference (*P* = 0.811) (Table 3). Postoperative AE did not occur in patients who had neither IIP nor AD‑IP (Supplementary Table 2). The three patients with AD-IP who developed IP-AE had Hashimoto’s disease, rheumatoid arthritis, and systemic sclerosis.

**Table 4:** Comparison of risk factors for IP-AE

Factors		IIP (n = 57)	AD-IP (n = 25)	*P-*value
Sex	Female	7 (12.3%)	13 (52.0%)	< 0.001
	Male	50 (87.7%)	12 (48.0%)	
Preoperative steroid	No	55 (96.5%)	13 (52.0%)	< 0.001
	Yes	2 (3.5%)	12 (48.0%)	
History of AE	No	57 (100%)	25 (100%)	–
	Yes	0 (0%)	0 (0%)	
KL-6 (U/ml)	≤ 1000	47 (82.5%)	20 (80.0%)	0.791
	> 1000	10 (17.5%)	5 (20.0%)	
%VC (%)	> 80%	53 (93.0%)	16 (64.0%)	< 0.001
	≤ 80%	4 (7.0%)	9 (36.0%)	
UIP pattern	No	46 (80.7%)	17 (68.0%)	0.210
	Yes	11 (19.3%)	8 (32.0%)	
Surgical procedure	Wedge resection	12 (21.1%)	7 (28.0%)	0.493
	≥ segmentectomy	45 (78.9%)	18 (72.0%)	

*AD* autoimmune disease, *AE* acute exacerbation, *IIP* idiopathic interstitial pneumonia, *IP* interstitial pneumonia, *KL-6* Klebs von den Lungen-6, *UIP* usual interstitial pneumonia, *VC* vital capacity

## Discussion

In the present study, among patients with lung cancer, those with IIP and AD-IP had poorer postoperative outcomes following surgery than did those with AD alone or neither condition. The poorer survival in patients with IP may be explained by its association with more aggressive pathological features, such as higher pathological stage and squamous cell histology, consistent with previous reports [15]. Similarly, the worse prognosis observed in patients with lung cancer who had AD after surgery was in line with the previous report [4], supporting the credibility of our dataset for analyzing clinicopathological features and survival outcomes.

Patients with AD-IP were more likely to be female, be younger, and have a lower Brinkman index—factors usually linked to a better prognosis—compared with patients who had IIP [16–18]. Additionally, no significant differences in pathological characteristics associated with a poor prognosis were observed between IIP and AD-IP. Nonetheless, patients with AD-IP demonstrated a tendency toward shorter RFS and OS than patients with IIP, consistent with the results reported by Maeda et al. [19]. One possible explanation might be the higher frequency of preoperative steroid use in patients with AD-IP than in those with IIP, which may promote tumor progression by creating an immunosuppressive microenvironment, as glucocorticoids are known to induce immunosuppression [20]. However, this possible association was not verified immunologically or pathologically in this study. These findings suggest that careful attention appears warranted in the follow-up of patients with AD-IP after surgical resection.

In our cohort, the frequency of IP-AE after surgery was 10.3%, which is very similar to that reported in a large-scale Japanese analysis (9.3%) [2]. Intriguingly, the incidence of IP-AE in patients with AD-IP was comparable to that in patients with IIP (11.5% vs. 9.8%, *P* = 0.811). Suda et al. reported that the 1-year and overall incidence of IP-AE in 83 patients with AD-IP after diagnosis were 1.3% and 7.5%, respectively [8]. Likewise, Park et al. found a 1-year frequency of 3.3% in 93 patients with surgical lung biopsy confirmed AD-IP [9]. Their frequencies were much lower than in the present study, most likely because these reports did not include patients with lung cancer and AD-IP undergoing surgery. Maeda et al. also reported that among 70 patients with lung cancer who had IIP and 16 who had AD-IP, the rates of IP-AE were 8.6% (6/70) and 6.3% (1/16), respectively (*P* = 0.759) [19]. In our cohort, the incidence of IP-AE in AD-IP was nearly twice that reported by Maeda et al. (11.5% vs. 6.3%). This difference may be explained by the higher rate of lobectomy in our study (77% vs. 50%). Also, the rate of AE might have been affected by different underlying ADs: our cohort included a broader spectrum of ADs (Supplementary Table 1), while the cohort of Maeda et al. included only four types, which included only four types—rheumatoid arthritis, Sjögren syndrome, dermatomyositis, and systemic lupus erythematosus. Nonetheless, both studies involved relatively small numbers of patients with AD-IP (26 in our study vs. 16 in Maeda et al.), and future studies with larger cohorts will be necessary to clarify the true incidence of IP-AE after surgical lung resection.

Despite the similar frequencies of IP-AE between IIP and AD-IP, the distribution of factors included in the risk score for IP-AE differed between IIP and IP-AE: male sex appeared to be less frequent among patients with AD-IP, while preoperative steroid use and %VC of ≤ 80% appeared to be more frequent. This suggests that AD-IP may represent a distinct entity from IIP despite the fact that both are classified as IP. A large-scale Japanese analysis of IP-AE after pulmonary resection for lung cancer reported that 5.8% (102/1763) of cases were AD-IP; however, it remains unclear how many patients with AD-IP were included in the study that established the IP-AE risk score based on 1022 patients [2, 14]. This highlights the need for a risk score specific to AD-IP. In our study, the three patients with AD-IP who developed IP-AE had Hashimoto’s disease, rheumatoid arthritis, and systemic sclerosis. By contrast, Maeda et al. did not provide detailed information on the type of AD in the one patient with AD-IP who developed postoperative IP-AE [19]. Taken together, our results suggest that all types of AD-IP should be carefully monitored for IP-AE after pulmonary resection.

The present study has two main limitations. First, its retrospective and single-center design may have introduced selection bias. Second, although the overall cohort exceeded 1000 patients, the number of patients with AD-IP was small (n = 26). Third, the AD-IP group comprised patients with various underlying AD. Grouping these distinct conditions together as AD-IP might obscure important differences because their pathophysiology and prognosis differ substantially. Larger studies with more patients are needed to clarify survival differences between IIP and AD-IP, determine the true frequency of IP-AE after surgical lung resection, and assess the validity of the IP-AE risk score.

## Conclusion

In the present study, lung cancer with AD-IP had a poor prognosis, as did lung cancer with IIP, and the frequency of postoperative IP-AE was similar between the two groups. Although future studies with more patients with AD-IP are needed, these findings may help guide the postoperative management of patients with lung cancer and AD-IP after pulmonary resection.

## Supplementary Information

Below is the link to the electronic supplementary material.

Supplementary Material 1

Supplementary Fig. 1 Survival following surgery. (A) Recurrence-free survival and (B) overall survival in the entire cohort of 1,281 patients with lung cancer

Supplementary Fig. 2 Survival following surgery. Lung cancer–specific overall survival of patients with IIP, AD without IP, AD-IP, and patients without these conditions (log-rank test: P < 0.001). AD, autoimmune disease; AD-IP, autoimmune disease–related interstitial pneumonia; IIP, idiopathic interstitial pneumonia; IP, interstitial pneumonia
