# Supplementary material for: Surgical outcomes of lung cancer associated with autoimmune disease-related interstitial pneumonia
Source: Gen Thorac Cardiovasc Surg. 2025 Nov 21;74(5):518–26. doi: 10.1007/s11748-025-02229-9 (PMC13139268; doi:10.1007/s11748-025-02229-9)
Supplement: Supplementary file 1 — Supplementary Material 1 [file 11748_2025_2229_MOESM1_ESM.docx]

**Supplementary Table 1.** Detailed information on AD.

| **Disease** | **AD without IP (n = 87)** | **AD-IP (n = 26)** |
| --- | --- | --- |
| Rheumatoid arthritis | 17 (19.5%) | 8 (30.8%) |
| Systemic sclerosis | 7 (8.0%) | 9 (34.6%) |
| Sarcoidosis | 5 (5.7%) | 1 (3.8%) |
| Sjögren’s syndrome | 3 (3.4%) | 0 (0.0%) |
| Systemic lupus erythematosus | 4 (4.6%) | 0 (0.0%) |
| IgG4-related disease | 1 (1.1%) | 0 (0.0%) |
| Behçet’s disease | 2 (2.3%) | 0 (0.0%) |
| Adult Still’s disease | 2 (2.3%) | 0 (0.0%) |
| Hashimoto’s disease | 11 (12.6%) | 1 (3.8%) |
| Graves’ disease | 3 (3.4%) | 0 (0.0%) |
| Type 1 diabetes | 2 (2.3%) | 0 (0.0%) |
| Primary biliary cirrhosis | 3 (3.4%) | 0 (0.0%) |
| Ulcerative colitis | 2 (2.3%) | 0 (0.0%) |
| Autoimmune hepatitis | 1 (1.1%) | 0 (0.0%) |
| Psoriasis | 2 (2.3%) | 0 (0.0%) |
| Pemphigoid | 1 (1.1%) | 0 (0.0%) |
| Pemphigus | 1 (1.1%) | 0 (0.0%) |
| Polymyalgia rheumatica | 4 (4.6%) | 0 (0.0%) |
| Polymyositis/dermatomyositis | 1 (1.1%) | 1 (3.8%) |
| Aortitis | 3 (3.4%) | 0 (0.0%) |
| Antiphospholipid syndrome | 1 (1.1%) | 0 (0.0%) |
| Panhypopituitarism | 1 (1.1%) | 0 (0.0%) |
| ANCA-associated vasculitis | 1 (1.1%) | 3 (11.5%) |
| Minimal change nephrotic syndrome | 1 (1.1%) | 0 (0.0%) |
| Eosinophilic granulomatosis with polyangiitis | 1 (1.1%) | 0 (0.0%) |
| Isolated ACTH deficiency | 0 (0.0%) | 1 (3.8%) |
| Cushing’s syndrome | 1 (1.1%) | 0 (0.0%) |
| Psoriatic arthritis | 1 (1.1%) | 0 (0.0%) |
| Erythroderma | 1 (1.1%) | 0 (0.0%) |
| Rheumatoid arthritis and systemic lupus erythematosus | 1 (1.1%) | 1 (3.8%) |
| Sjögren’s syndrome and primary biliary cirrhosis | 1 (1.1%) | 0 (0.0%) |
| Rheumatoid arthritis and aortitis | 1 (1.1%) | 0 (0.0%) |
| Polyarteritis and Sjögren’s syndrome | 1 (1.1%) | 0 (0.0%) |
| Polymyositis and antiphospholipid syndrome | 0 (0.0%) | 1 (3.8%) |

ANCA, antineutrophil cytoplasmic antibody; ACTH, adrenocorticotropic hormone; AD, autoimmune disease; AD-IP, autoimmune disease–related interstitial pneumonia; IgG4, immunoglobulin G4.

**Supplementary Table 2.** Comparison of perioperative outcomes in patients with IIP, AD without IP, AD-IP and patients without these conditions.

| **Characteristics** |  | **IIP (n = 61)** | **AD without IP (n = 87)** | **AD-IP (n = 26)** | **Without any**  **(n = 1,107)** | ***P-*value** |
| --- | --- | --- | --- | --- | --- | --- |
| Postoperative hospital stay (days) | Median (IQR) | 10 (7, 14) | 7 (6, 10) | 11 (8, 18) | 8 (6, 11) | < 0.001 |
| Operation time (min) | Median (IQR) | 185 (120, 242) | 200 (138, 289) | 152 (105, 242) | 206 (152, 261) | 0.158 |
| Blood loss (mL) | Median (IQR) | 100 (25, 216) | 65 (7, 120) | 110 (0, 165) | 80 (17, 150) | 0.465 |
| Re-operation | N (%) | 2 (3.3%) | 3 (3.5%) | 3 (11.5%) | 41 (3.7%) | 0.229 |
| Cardiovascular | N (%) | 2 (3.3%) | 5 (5.8%) | 0 (0.0%) | 44 (4.0%) | 0.602 |
| Bronchopulmonary fistula | N (%) | 0 (0.0%) | 0 (0.0%) | 21 (3.9%) | 5 (0.45%) | 0.070 |
| Pneumonia | N (%) | 5 (8.2%) | 5 (5.8%) | 5 (19.2%) | 60 (5.4%) | 0.025 |
| Empyema | N (%) | 3 (4.9%) | 3 (3.4%) | 1 (3.9%) | 30 (2.7%) | < 0.001 |
| Prolonged air leak | N (%) | 11 (18.0%) | 5 (5.8%) | 3 (11.5%) | 84 (7.6%) | 0.023 |
| AE of IP | N (%) | 6 (9.8%) | 0 (0.0%) | 3 (11.5%) | 0 (0.0%) | < 0.001 |
| Postoperative death within 30 days | N (%) | 1 (1.64%) | 0 (0.0%) | 0 (0.0%) | 1 (0.09%) | 0.028 |

AD, autoimmune disease; AE, acute exacerbation; IIP, idiopathic interstitial pneumonia; IP, interstitial pneumonia; IQR, interquartile range.
