# Supplementary figures and images for: Surgical outcomes of lung cancer associated with autoimmune disease-related interstitial pneumonia
Source: Gen Thorac Cardiovasc Surg. 2025 Nov 21;74(5):518–26. doi: 10.1007/s11748-025-02229-9 (PMC13139268; doi:10.1007/s11748-025-02229-9)

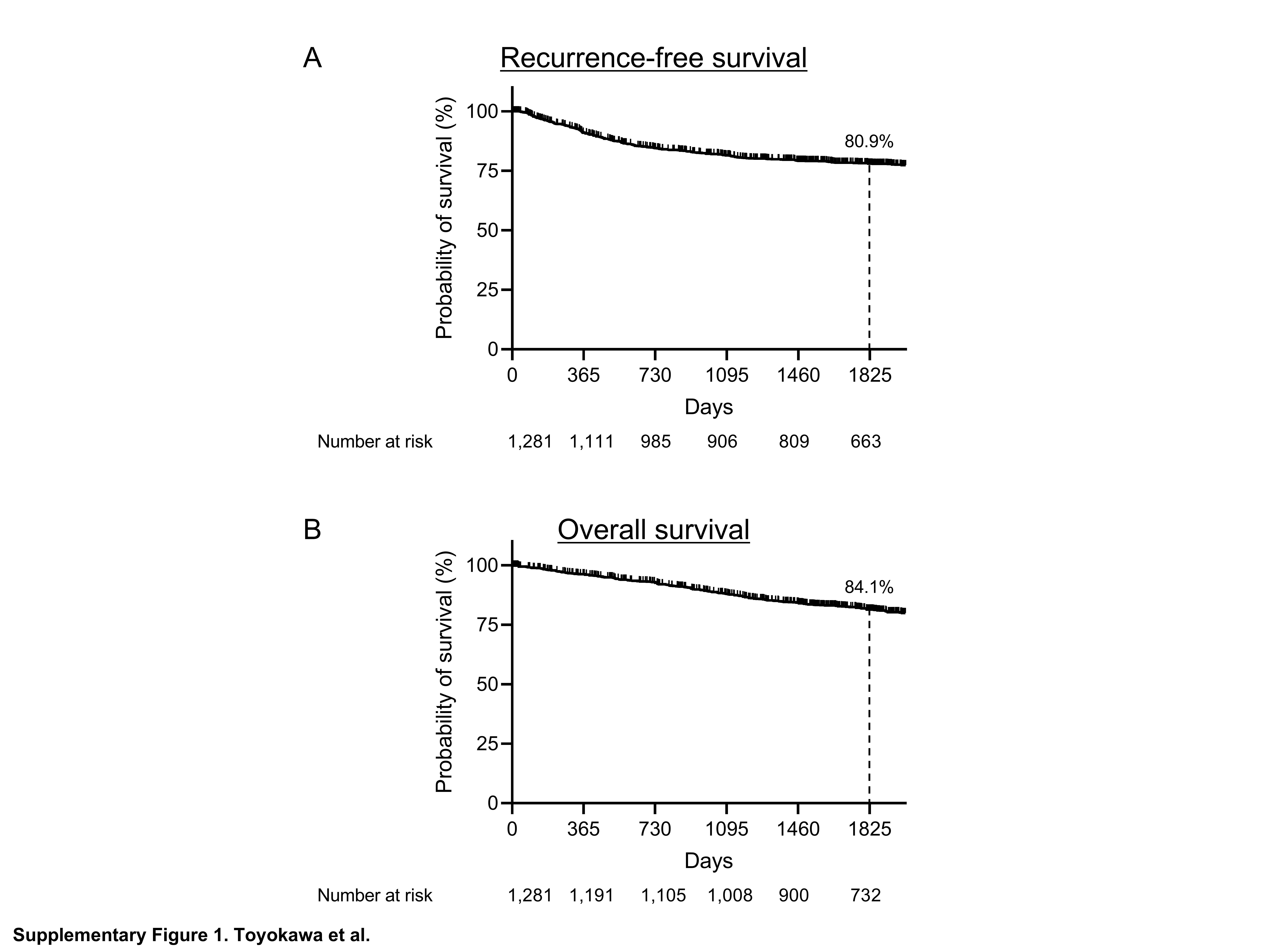

Supplement: Supplementary file 2 — Supplementary Fig. 1 Survival following surgery. (A) Recurrence-free survival and (B) overall survival in the entire cohort of 1,281 patients with lung cancer [file 11748_2025_2229_MOESM2_ESM.tif]

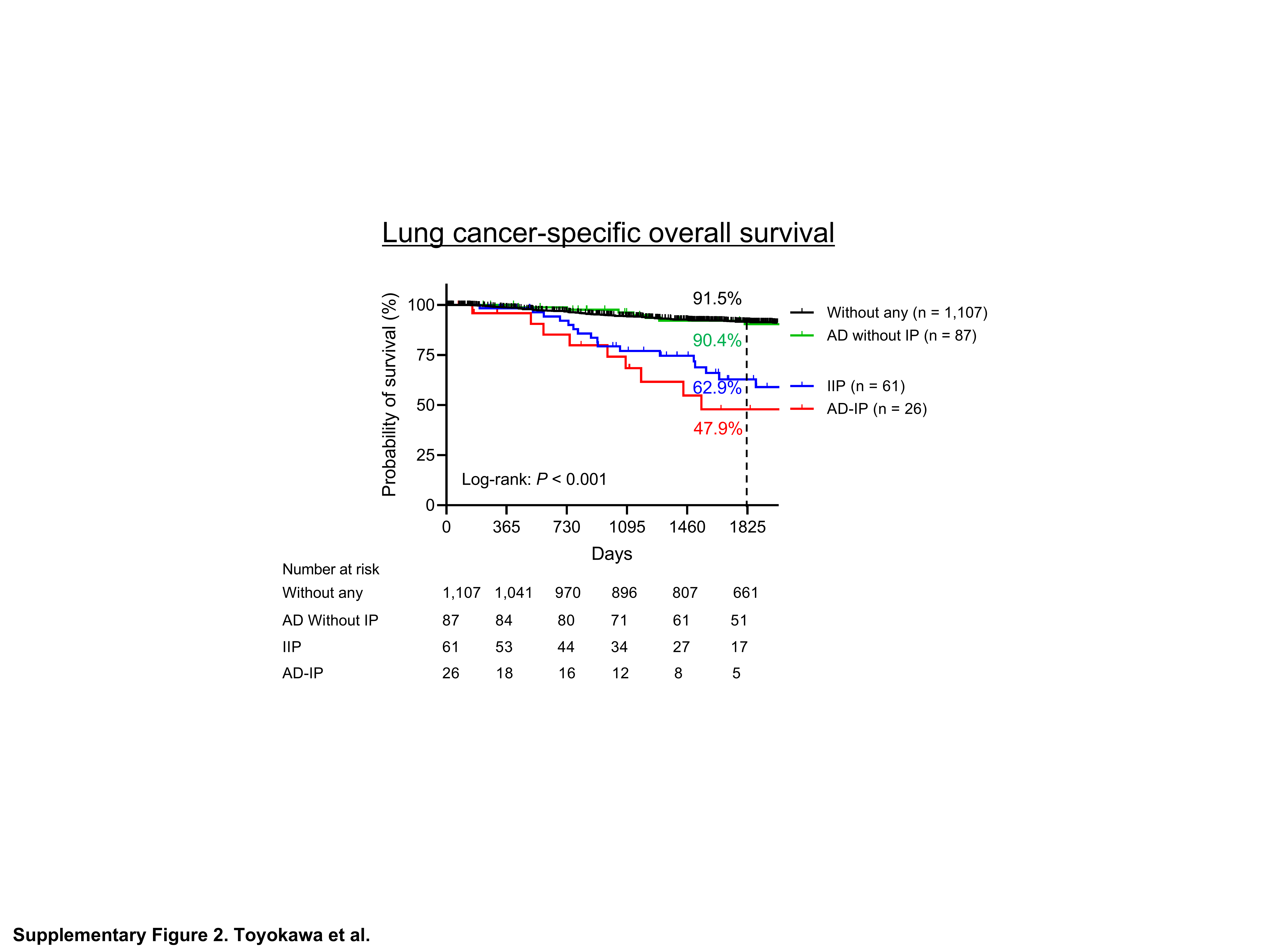

Supplement: Supplementary file 3 — Supplementary Fig. 2 Survival following surgery. Lung cancer–specific overall survival of patients with IIP, AD without IP, AD-IP, and patients without these conditions (log-rank test: P < 0.001). AD, autoimmune disease; AD-IP, autoimmune disease–related interstitial pneumonia; IIP, idiopathic interstitial pneumonia; IP, interstitial pneumonia [file 11748_2025_2229_MOESM3_ESM.tif]
